# Socio-economic inequalities in children’s nutritional status in Democratic Republic of the Congo in 2017–2018: an analysis of data from a nationally representative survey

**DOI:** 10.1017/S1368980021004249

**Published:** 2022-02

**Authors:** Xinran Qi, Yifan Zhu, Yu Wang, Qiwei He, Jiayi Hee, Wei Chen, Rie Takesue, Kun Tang

**Affiliations:** 1 Vanke School of Public Health, Tsinghua University, No. 30 Shuangqing Road, Beijing 100084, People’s Republic of China; 2 School of Nursing, Capital Medical University, Beijing, People’s Republic of China; 3 Xiangya School of Medicine, Central South University, Changsha, People’s Republic of China; 4 Bloomberg School of Public Health, Johns Hopkins University, Baltimore, USA; 5 School of Management, Ocean University of China, Qingda, People’s Republic of China; 6 School of Medicine, Tsinghua University, Beijing, People’s Republic of China; 7 School of Public Health, The University of Queensland, Brisbane, Australia; 8 Health Section Programme Division, UNICEF Headquarters, New York, USA

**Keywords:** Socio-economic inequality, Children’s nutritional status, Maternal healthcare coverage, Democratic Republic of Congo

## Abstract

**Objective:**

The Democratic Republic of the Congo (DRC) has one of the highest levels of child undernutrition globally; however, little information exists on the underlying socio-economic inequalities resulting in undernutrition. This study aims to examine the differences in the nutritional statuses of children across different wealth quintiles and explores the association between malnutrition in children and related factors.

**Design:**

We utilised the 2018 Multiple Indicator Cluster Survey data. We estimated the prevalence of malnutrition across all twenty-six provinces. The study used the WHO 2006 child growth standards to measure stunting, underweight and wasting. We employed a mixed-effect linear model to analyse the association between nutritional status and healthcare accessibility, domestic sanitation, and socio-demographic factors.

**Setting:**

Twenty-six provinces in the DRC.

**Participants:**

21 477 children under 5 years of age and 21 828 women of childbearing age in the DRC.

**Results:**

The national prevalence of underweight, stunting and wasting was found to be 23·33 %, 42·05 % and 5·66 %, respectively. Household wealth and mother’s education level were significantly positively associated with the nutritional statuses of children. Among households in the lowest wealth quintile, residence in urban areas was a protective factor against undernutrition.

**Conclusion:**

The findings of this study indicate considerable socio-economic inequalities in the nutritional statuses of children under 5 years of age in the DRC, highlighting the need for nutrition promotion as part of maternal and child healthcare. Interventions and policies should include improving nutrition education for less-educated mothers, in particular, in the central provinces of the DRC.

The Democratic Republic of the Congo (DRC) is the largest country in sub-Saharan Africa with a population of over 84 million people (2018)^(1)^. The macroeconomic and financial development of the DRC has been rapidly increasing since 2001. The gross domestic product (GDP) of the DRC increased from 7·4 billion dollars to 50·4 billion dollars in 2019^(2)^, making the DRC one of the fastest-growing economies in the world^(3)^. Despite the significant economic growth and abundance of mineral and agricultural resources, large disparities between socio-economic statuses continue to exist in the DRC. According to data from the World Bank, 73 % of the Congolese population, or approximately 60 million people in 2018, lived on less than $1·90 a day, and one out of six people lived in extreme poverty^(4)^. Over the past decade, the DRC has been affected by waves of civil wars and unceasing domestic conflicts. Violence, mass displacement and the destruction of infrastructure have devastated the health of the population^(5,6)^. As of 2008, an estimated 5·4 million deaths occurred as a result of the armed conflict between 1998 and 2007^(7)^, mainly due to disease and starvation^(8)^.

The DRC has among the highest percentages of chronic child malnutrition, or stunting, in the world with lasting consequences for the children who suffer from this nutritional disorder^(9)^. Malnutrition during early childhood is associated with adverse effects on cognitive and physical development^(10,11)^ and failure of proper development during the ‘critical age window of 1000 days’ can lead to cognitive and physical deficits in adulthood^(12,13)^. This can in turn result in poorer educational and economic outcomes^(14–16)^. According to the 2018 Human Capital Index (HCI) which measures the survival of children using key health determinants, the DRC ranked 146 out of 157 countries^(17)^, indicating high mortality rates in children in the DRC.

Apart from the effects of malnutrition on stunting, studies have examined the association between factors such as socio-economic inequalities due to unequal distribution of power, wealth and resources with stunting in children^(18–21)^. Higher educational attainments of parents and place of residence were found to be positively related to the nutritional statuses of children^(19,22–24)^. However, there is a need for studies to further explore the risk of stunting at different levels of determination to identify key factors contributing to the progression of stunting in the DRC, especially at a national level. For this study, a conceptual framework for determinants of malnutrition was utilised to compare the importance of determinants (e.g. geographical and socio-economic indicators) at different levels and their impacts on household income and health service inequalities in the DRC^(25)^. The findings of this study aim to provide decision-makers with comparative information on the impact of different factors affecting the nutritional statuses of children to provide empirical and evidence-based guidance to inform future interventions and policies^(20,26–29)^.

## Methods

### Data

Data from the 2018 Multiple Indicator Cluster Survey (MICS-Palu) were utilised for this study. The MICS is a nationally representative household survey of children aged 0–5 years, women aged 15–49 years and men aged 15–59 years. This survey was conducted by the National Institute of Statistics and the Ministry of Planning of the DRC, in collaboration with the UNICEF and the United States Agency for International Development/Centres for Disease Control (USAID/CDC). The MICS generated more than 150 key indicators that provided disaggregated data on the situation of children and women, and the living situations of the population in the areas of health, nutrition, education, security, hygiene and sanitation.

Multi-stage stratified cluster sampling was employed for the selection of the survey sample. Both urban and rural areas across all twenty-six provinces in the DRC were included. Additionally, urban and rural areas were identified as the main sampling strata using a two-stage selection method. The urban and rural areas within each province were identified as the main sampling strata, and the sample of households was selected in two stages. Within each stratum, 721 clusters were selected systematically with probability proportional to size. After a household listing was carried out within the cluster, a systematic sample of thirty households was drawn in each cluster.

### Variables

Height-for-age *Z* score (HAZ), weight-for-age *Z* score (WAZ) and weight-for-height *Z* score (WHZ) were analysed using the 2006 WHO child growth standards^(30)^. A plausibility criterion of –6 to 6 sd from the median of the reference population has been used for WAZ and WHZ, and –7 to 7 sd from the median of the reference population for HAZ. We defined stunting as HAZ more than 2 sd below the median of the reference population, underweight as WAZ more than 2 sd below the median, wasting as WHZ more than 2 sd below the median and overweight as WHZ more than 2 sd above the median.

Four outcomes of maternal healthcare were selected for our study: delivery at a healthcare facility (public/private), antenatal care coverage (defined as four or more antenatal care visits), skilled attendants at birth and postnatal care (defined as a health check-up while in the facility or at home following delivery, or a postnatal care visit within 2 d after delivery).

Handwashing behaviours, clean water sources and improved toilet facilities were selected to analyse household sanitation. Correct handwashing behaviour was assessed using the availability of proper handwashing facilities, clean water and soap. Improved sources of drinking water were assessed with the use of the following types of water supply: piped water, tube well/borehole, protected dug well, protected spring, rainwater collection, and packaged or delivered water. Improved toilet facilities were assessed with the use of flush or pour flush to piped sewer systems, septic tanks, or pit latrines, ventilated improved pit latrines, pit latrines with slabs, and composting toilets.

Household economic status (measured using the wealth index) was utilised to measure health inequalities. Principal components analysis was performed to construct the wealth index by using the information on the ownership of consumer goods, dwelling characteristics and other characteristics that were related to household’s wealth, to generate weights (factor scores) for each of the items used^(31)^. Highest educational attainment of mothers (no formal education or pre-school, primary school, secondary school, and tertiary and above), child’s weight at birth, age (in months), sex (male or female) and place of residence (rural or urban) were also taken into account.

### Statistical analysis

Univariable and multivariable associations between the nutritional statuses of children and individual determinants such as the child’s age and sex, mother’s education level, maternal healthcare including the use of qualified birthing facilities, antenatal visits of more than four times, skilled attendants at birth, postnatal care checks, and household determinants including household wealth index and place of residence (urban *v*. rural) were analysed. The covariates were chosen based on previous literature on malnutrition in developing countries^(32,33)^. The mixed-effects model with a random intercept specified at the primary sampling unit level was used to adjust for unmeasured factors that might affect the nutritional status of children. These features of the regression model also account for complex survey design. All the children included in the model were matched to their mother by ‘Cluster number, Household number and Mother’s line number’ provided by the original questionnaires for both adults and children.

## Results

### Descriptive characteristics

The characteristics of the participants are presented in Table 1. A total of 21 456 children under 5 years of age and 21 756 women of childbearing age in the DRC were analysed for this study. Among the 21 456 children under 5 years of age, the participants had a mean age of 2·44 years, a mean HAZ, WAZ and WHZ of –1·6, –1·05 and –0·15, respectively. The national prevalence rate of underweight, stunting and wasting was found to be 23·33 %, 42·05 %, and 6·54 %, respectively, whilst the prevalence rate of overweight was 3·80 %. The majority of children resided in rural areas (58·42 %). The majority of mothers had either no formal/pre-school education or primary school education (53·06 %). For the maternal healthcare coverage, the utility rate of antenatal visits over four times, delivery at health facilities, skilled attendants at birth and postnatal health checks were 43·21 %, 81·53 %, 85·20 %, and 56·59 %, respectively. In terms of domestic sanitation, the coverage of handwashing facilities, improved toilet and improved water were 56·73 %, 28·77 % and 54·66 %, respectively.

**Table 1:** Descriptive statistics for main variables in the analysis

	Mean	Proportion (%)	sd/se
Child’s age (months)	29·35		0·15
Woman’s age (years)	29·08		0·12
Height-for-age *Z* score	−1·60		0·04
Weight-for-age *Z* score	−1·05		0·02
Weight-for-height *Z* score	−0·15		0·02
Weight at birth (kg)	3·33		0·01
Household size	6·74		0·05
Child’s sex			
Male		49·34	0·50
Female		50·66	0·50
Stunting		42·05	1·01
Underweight		23·33	0·73
Wasting		6·54	0·41
Overweight		3·80	0·26
Mother’s education level			
Illiteracy		18·91	1·06
Primary school		34·15	1·06
Secondary school 1		14·28	0·72
Secondary school 2		29·35	1·10
Above		3·30	0·43
Location of residence			
Urban		41·58	2·57
Rural		58·42	2·57
Coverage of maternal healthcare			
Antenatal visit over four times		43·21	1·33
Delivery at health facility		81·53	1·20
Skilled attendants at birth		85·20	1·16
Postnatal health checks		56·59	1·41
Domestic sanitation			
Handwashing		56·73	2·12
Improved toilet		28·77	1·84
Improved water		54·66	2·57

### Factors associated with children’s nutritional status – socio-economic inequalities

**Fig. 1:**
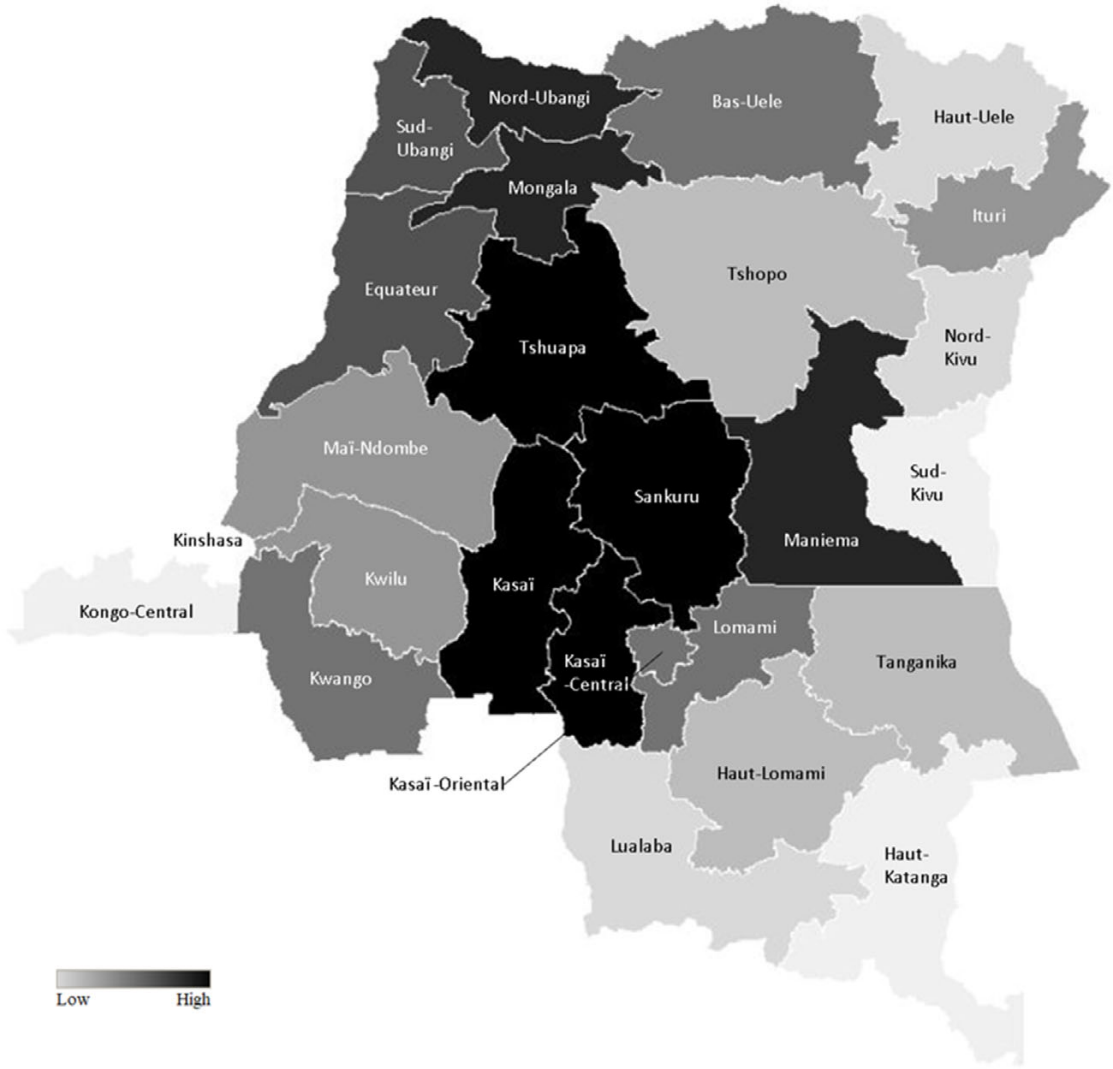
Distribution of the lowest wealth quintile households across the DRC. DRC, The Democratic Republic of the Congo

Figure 1 illustrated the distribution of wealth across all twenty-six provinces in the DRC. Households in the lowest wealth quintile are concentrated among the central provinces of the DRC and account for 46 %–60 % of households whereas, in western and southeastern provinces such as Kongo Central and Haut-Katanga, households in the lowest wealth quintile accounted for a smaller proportion (9 %–15 % of households). In Kinshasa, however, the proportion of households in the lowest wealth quintile was below 9 %.

**Fig. 2:**
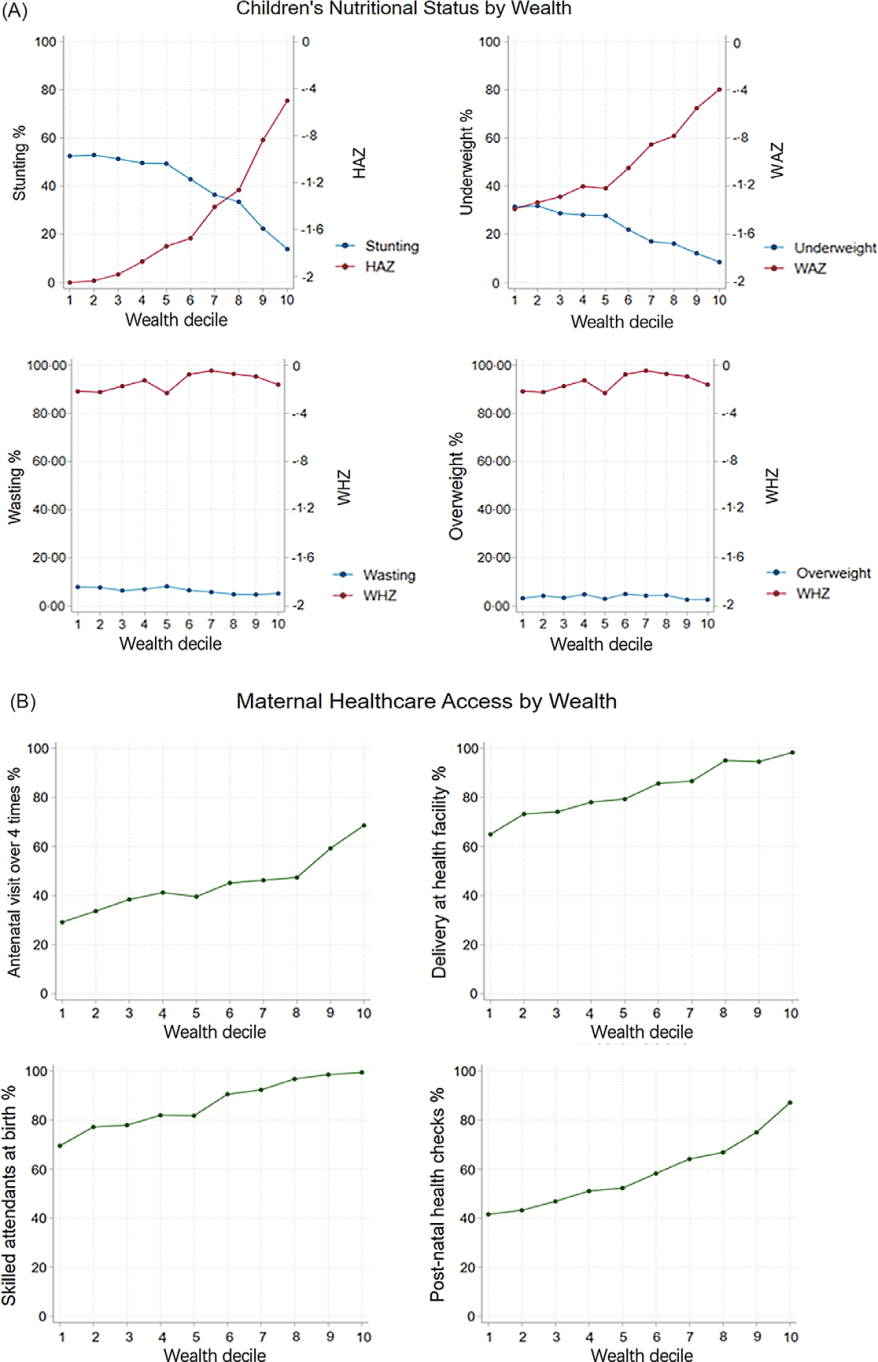
The children’s nutritional status and maternal healthcare indicators according to decile of wealth index

Nutrition status among children and maternal healthcare access differed across wealth quintiles. Higher household economic status was protective against children’s stunting (HAZ) and underweight (WAZ). However, such a protective effect was not found among wasting and overweight (WHZ) in Fig. 2. Therefore, children’s nutritional status was mainly assessed by WAZ and HAZ later in this study. Maternal healthcare, including the percentage of mothers with access to qualified birthing locations, antenatal care, skilled attendants at birth and postnatal care, was positively associated with the increasing household wealth (from the lowest to the highest wealth quintile) (Fig. 2).

After matching, there were 20 202 mother–child dyads. Among them, 10 959 mothers had given birth within 2 years before the investigation and thus had information on maternal healthcare and were included in the regression analysis. Results from the crude random intercept linear regression estimating the association of the nutritional status of children with mother’s maternal healthcare and household socio-demographic factors indicate that among children, HAZ and WAZ were positively associated with maternal healthcare and domestic sanitation (Table 2). However, when employing a multivariable-adjusted regression, there was no statistically significant association between the nutritional status of children and maternal healthcare or domestic sanitation. The wealth index appeared to be positively associated with HAZ and WAZ. Likewise, mothers’ education level was also positively associated with HAZ and WAZ among children. Average WAZ was higher among children whose mothers had secondary level schooling, secondary school and above compared to those whose mothers had no formal schooling.

**Table 2:** Crude and adjusted associations of the nutritional status of children with maternal healthcare for the mother and household socio-demographic factors

	HAZ						WAZ						WHZ					
	Crude			Multivariable-adjusted			Crude			Multivariable-adjusted			Crude			Multivariable-adjusted		
	Coefficient	95 % CI	*P* value	Coefficient	95 % CI	*P* value	Coefficient	95 % CI	*P* value	Coefficient	95 % CI	*P* value	Coefficient	95 % CI	*P* value	Coefficient	95 % CI	*P* value
Maternal healthcare index	0·029	–0·007, 0·065	0·113	0·010	–0·025, 0·046	0·567	0·036	0·009, 0·063	0·009	0·024	–0·003, 0·050	0·084	0·024	–0·003, 0·052	0·085	0·022	–0·006, 0·050	0·117
Domestic sanitation index	0·051	0·005, 0·097	0·029	−0·031	–0·078, 0·015	0·189	0·030	–0·004, 0·064	0·081	−0·018	–0·053, 0·017	0·318	0·003	–0·032, 0·038	0·884	0·001	–0·036, 0·038	0·963
Wealth index				0·257	0·198, 0·315	<0·001				0·145	0·101, 0·189	<0·001				−0·009	–0·055, 0·037	0·713
Mother’s education (reference: illiteracy)																		
Primary school				0·097	0·006, 0·187	0·037				0·101	0·034, 0·169	0·003				0·065	–0·005, 0·134	0·070
Secondary school 1				0·237	0·122, 0·352	<0·001				0·220	0·134, 0·306	<0·001				0·123	0·034, 0·211	0·007
Secondary school 2				0·223	0·112, 0·333	<0·001				0·213	0·130, 0·295	<0·001				0·108	0·023, 0·193	0·013
Above secondary school				0·410	0·147, 0·674	0·002				0·334	0·137, 0·531	0·001				0·151	–0·052, 0·354	0·144

HAZ, height-for-age *Z* score; WAZ, weight-for-age *Z* score; WHZ, weight-for-height *Z* score.

Estimations from all models are adjusted for the place of residence (rural/urban), household size, child’s age (months) and sex, and mother's age (years).

### Factors associated with children’s nutritional status and maternal healthcare access geographical inequalities

**Fig. 3:**
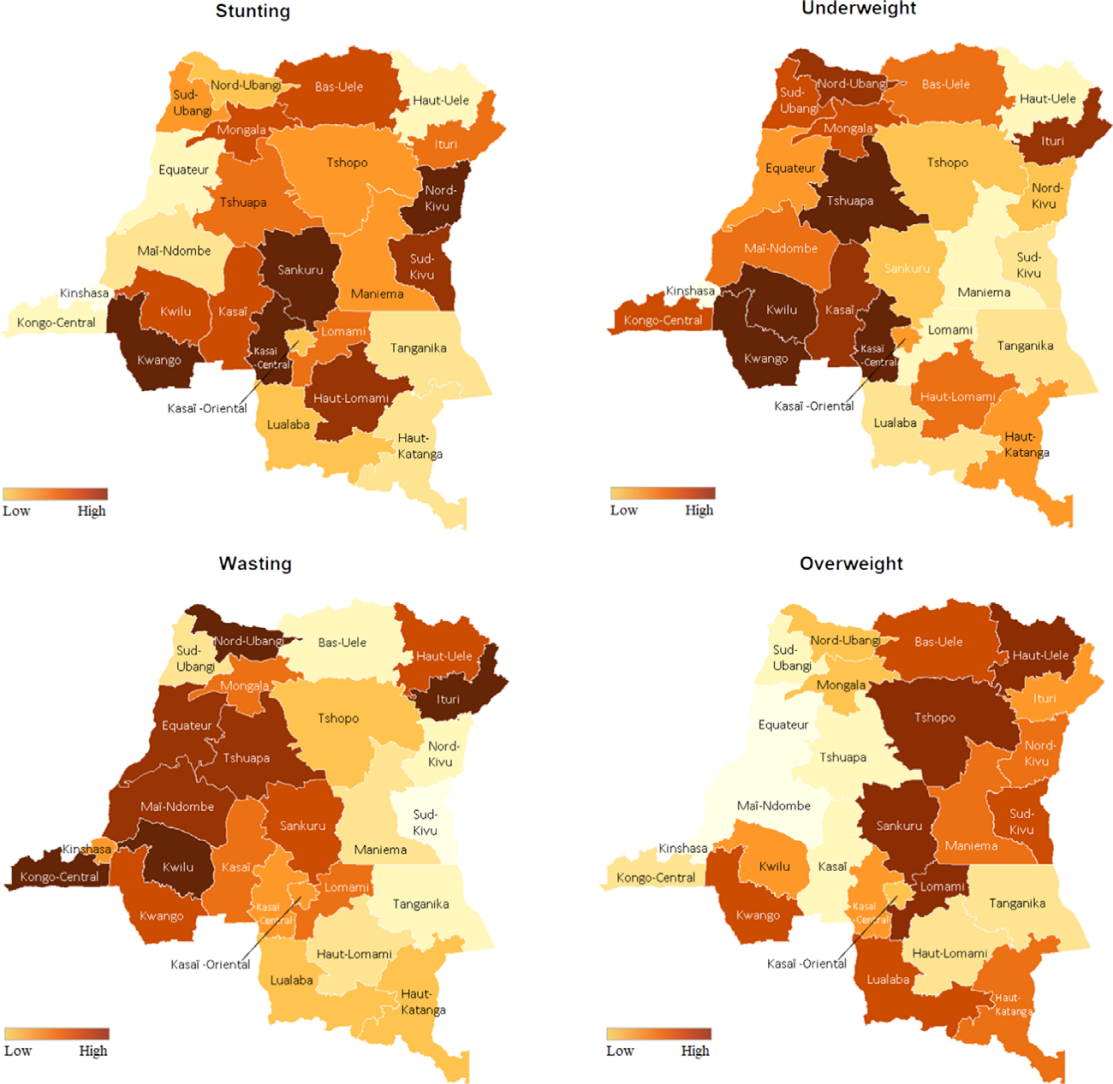
The prevalence of underweight, stunting, wasting and distribution of economic status across the country

Figure 3 demonstrated province-specific net spatial effects of nutrition status among children, including the prevalence of underweight, stunting, wasting and overweight in the DRC. Darker colours correspond to higher prevalence, whereas lighter colours correspond to lower prevalence. Our mapping exhibited three key observations. Firstly, the prevalence of malnutrition among children was generally higher among central provinces in the DRC. Secondly, southwest provinces, including Kwango, Kwilu, Kasaï and Kasaï-Central, exhibited a higher prevalence of stunting and underweight among children. Thirdly, taking into account that eastern provinces are subjected to ongoing conflict, nutrition status was poorer among children in these regions.

**Fig. 4:**
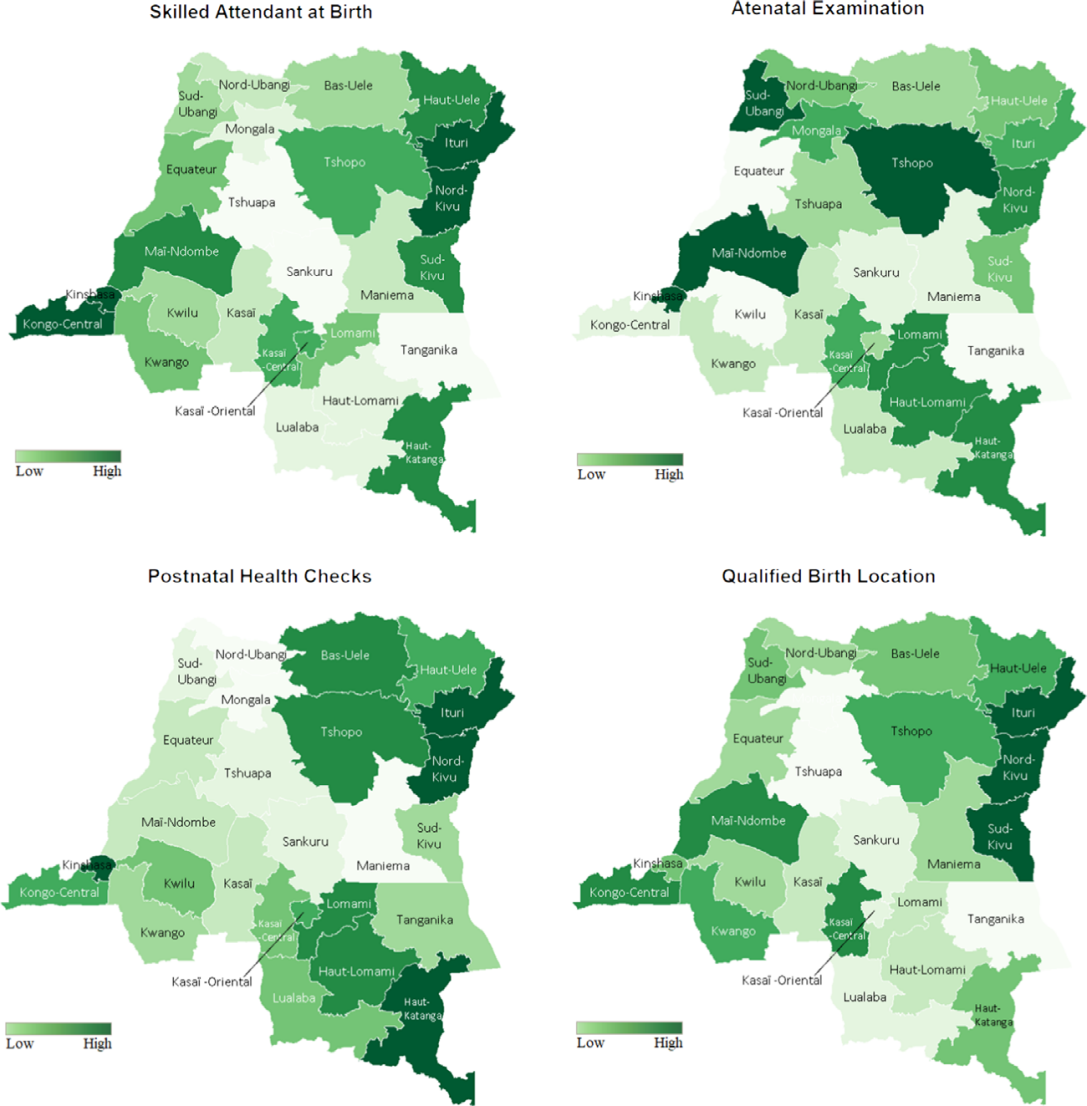
The coverage of qualified birth delivery location, antenatal care (ANC), skilled attendants at birth and postnatal care (PNC) among women

Maternal healthcare access coverage, including delivery at health facilities, antenatal care, skilled attendants at birth and postnatal care among women, is demonstrated in Fig. 4. Notably, western, eastern and southeastern provinces, such as Kongo Central, Kinshasa, and the Haut-Katanga, generally had good maternal healthcare conditions, in contrast to the lowest wealth quintile households being concentrated among the central provinces (Fig. 1). Additionally, it is worth noting that Kinshasa, the capital of the DRC, had both the best maternal healthcare and nutritional status among children in the country.

### Factors associated with children’s nutritional status (children’s height-for-age Z score and weight-for-age Z score) stratified by the lowest/highest wealth quintile in rural and urban settings

In the multivariable-adjusted analysis in Table 3, we stratified individuals from the lowest and highest wealth quintile and find that neither maternal healthcare index nor domestic sanitation index was associated with children’s HAZ and WAZ. Individuals falling within the lowest wealth quintile and living in rural areas had a lower HAZ (–0·585 (95 % CI –0·871, –0·300); *P* < 0·001) compared to those living in urban areas. Additionally, the mother’s education level was found to be a protective factor of children’s nutritional status. Average WAZ was higher among children whose mother had secondary school 2 (0·385 (95 % CI 0·161, 0·609); *P* = 0·001) and above secondary school (0·652 (95 % CI 0·222, 1·082); *P* = 0·003) education levels compared to children whose mothers had no schooling. Among the children falling within the highest wealth quintile, WAZ has a significant association with the wealth index only. On average, a one-score increase in wealth index was associated with a 0·347 increase in WAZ ((95 % CI 0·119, 0·576); *P* = 0·003). Furthermore, among those in the highest wealth quintile, there was no statistically significant association between the area of residence, mother’s education level and children’s nutritional status. Additionally, the result for the WHZ of children was also presented in the Appendix.

**Table 3:** Nutritional status of children from the households of the lowest and highest wealth quintile

	Lowest wealth quintile						Highest wealth quintile					
	HAZ			WAZ			HAZ			WAZ		
	Coefficient	95 % CI	*P* value	Coefficient	95 % CI	*P* value	Coefficient	95 % CI	*P* value	Coefficient	95 % CI	*P* value
Maternal healthcare Index	−0·038	–0·138, 0·062	0·455	−0·024	–0·099, 0·051	0·528	−0·020	–0·177, 0·137	0·800	0·046	–0·074, 0·166	0·452
Domestic sanitation index	−0·120	–0·255, 0·015	0·081	−0·008	–0·092, 0·076	0·852	−0·085	–0·212, 0·042	0·189	−0·034	–0·143, 0·075	0·541
Living in rural	−0·585	–0·871, –0·300	<0·001	−0·316	–0·515, –0·116	0·002	−0·356	–1·453, 0·741	0·525	0·480	–0·254, 1·214	0·199
Wealth index	0·112	–0·383, 0·607	0·657	0·276	–0·027, 0·578	0·074	0·265	–0·063, 0·593	0·114	0·347	0·119, 0·576	0·003
Mother’s education (reference: illiteracy)												
Primary school	−0·103	–0·349, 0·142	0·410	0·080	–0·108, 0·268	0·403	−0·191	–0·562, 0·179	0·311	−0·272	–0·578, 0·034	0·081
Secondary school 1	0·279	–0·048, 0·606	0·094	0·294	0·023, 0·565	0·034	0·147	–0·316, 0·609	0·534	−0·211	–0·578, 0·156	0·259
Secondary school 2	0·207	–0·118, 0·533	0·212	0·385	0·161, 0·609	0·001	0·015	–0·373, 0·403	0·940	−0·318	–0·641, 0·004	0·053
Above secondary school	2·314	0·730, 3·898	0·004	0·652	0·222, 1·082	0·003	0·022	–0·529, 0·573	0·937	−0·020	–0·470, 0·430	0·931

HAZ, height-for-age *Z* score; WAZ, weight-for-age *Z* score.

Estimations are also adjusted for household size, child’s age (months), weight at birth (kg), child’s sex, and mother’s age (years).

## Discussion

This study illustrates the DRC’s national profile of malnutrition among children between 2017 and 2018 using the nationally representative MICS survey. In comparison to the DRC’s MICS survey between 2010 and 2017^(34)^, the prevalence of stunting decreased from 43 % to 42 %, the prevalence of underweight decreased from 29 % to 23 % and the prevalence of wasting decreased from 7 % to 6·5 %, respectively. This trend is comparable to findings from the DRC’s 2013 Demographic Health Survey (DHS)^(34)^, where the prevalence of stunting, underweight and wasting were 43 %, 22·6 % and 7·9 %, respectively. In addition, a recent study that assessed malnutrition among children in forty-seven low-income and middle-income countries using 4–6 rounds of MICS^(35)^ between 2010 and 2017 found that the overall average rate of stunting, underweight and wasting was 32·5 %, 18·7 %, and 4·6 %, respectively. These rates are much lower than that of the DRC. Hence, malnutrition remains a significant health burden in the DRC.

This study also found a relationship between malnutrition, maternal healthcare coverage and the geographic distribution of provinces in the DRC. Central provinces in the DRC generally had a higher prevalence of malnutrition among children as well as lower coverage of maternal healthcare. This can be attributed to relatively unprosperous economies in these provinces (Fig. 1). Although these provinces have agricultural potential, due to the vast deposits of diamonds, people tend to rely on the artisanal mining industry for a living, instead of involving in agriculture^(36)^. The deterioration of the transportation network also poses negative impacts on food security^(37)^. Provinces in this region are landlocked with irregular train service in bad condition, which is difficult to reach. Furthermore, the violence that erupted in Kasaï during the August 2016 hindered the transportation of essential supplies, which negatively impacted the economy, causing price hikes on items such as food. As a consequence, hundreds of thousands of malnourished children were at increased risk of starvation^(36)^.

Over the years, the DRC’s Katanga region has been faced with chronic instability. However, children’s nutrition status and the coverage of maternal healthcare are relatively better in comparison to central provinces, which could be attributed to several factors. Firstly, the Katanga region is one of the three main economic hubs in the DRC with large commercial and industrial bases, highly developed mining industries, and agricultural sectors^(38)^. Secondly, vast investments and international health assistance have been concentrated in this region. For example, as of 2018, Doctors Without Borders run comprehensive medical programmes including maternal and paediatric health care in these provinces^(39)^. As a result, people residing in these regions are more likely to have access to healthcare resources as well as the motivation to utilise these services.

Our study further confirms the relationship between malnutrition and socio-economic inequalities. Results of the descriptive analysis show that as households’ wealth index scores increased, the prevalence of malnutrition decreases, while the coverage rate for maternal healthcare services increases. As Muennig and Su pointed out, money plays an important role in the health of individuals, especially in very poor countries where food is a critical element of survival and requires financial capabilities^(40)^. The random intercept linear regression model in our study (Table 2) confirms that the household’s wealth significantly improves the nutrition status of the children. The relationship between poverty and malnutrition has also been elaborated by Müller and Krawinkel, such that poverty leads to insufficient household food security, insufficient child and maternal care, and unhealthy environments^(41)^, which in turn cause malnutrition.

Historically, many studies in developing countries (such as Ethiopia, Indonesia and Bangladesh) have proved that community factors, such as domestic sanitation and healthcare service, are closely related to variations in children’s nutritional status^(42–44)^. Insufficient health services and an unhealthy environment are also important contextual factors that may contribute to worsening rates of malnutrition in the UNICEF framework of causes of malnutrition (UNICEF 1990)^(45)^. As demonstrated by the UNICEF framework, malnutrition is determined by a range of factors relying on the implementation of interventions by a multitude of sectors. Previous researches showed a strong relationship between the nutritional status of children and its immediate and underlying determinants. For example, in Gabon, feeding practices and access to improved water and sanitation were the best predictors of stunting^(46)^. In both Bangladesh and Vietnam, household food security, better caregiver nutrition knowledge and hygiene practices were positively associated with stunting^(47)^. However, results in Congo show that underlying causes such as domestic sanitation and maternal healthcare have little impact on children’s malnutrition while taking basic causes, namely economic structure into account. Using the principal component factor method, we identify the maternal healthcare index as a proxy for healthcare accessibility among households. To our surprise, the maternal healthcare index had no statistically significant association with children’s nutrition status after including household wealth score and mother’s education level. One potential explanation for this finding is that the child nutrition programme may have not been well integrated with maternal healthcare programmes in the DRC, as a result of a fragmented health system that is observed across and within disease control programmes. According to the country report from WHO, between 2006 and 2012, altogether 195 projects and programmes in the health sector of the DRC had external funding but lacked functional coordination among the different health programmes, even when housed in the same building^(48)^. In conclusion, even though maternal healthcare is accessible to households, it may not ensure the availability of child healthcare. Another possible explanation on why the maternal healthcare index has no impact on children’s nutrition status may be attributed to quality issues present in the healthcare delivery system. Since the 1990s, governmental funding for the public health sector has declined in the DRC, and health financing is highly accounted for by out-of-pocket payments (39 % in 2016^(8)^) and external aid (42·5 % in 2017^(8)^). With little public funding and weak national leadership, health sector regulation in the DRC has digressed. Additionally, in 2017, the infant mortality in the DRC was 43 per 1000 live births, the child mortality was 70 per 1000 births^(49)^ and the maternal mortality ratio was estimated at 693 per 100 000 live births in 2015^(50)^. Although healthcare accessibility exists, poor-quality healthcare at delivery may prohibit children from fully benefiting from these services.

To further assess the factors associated with children’s nutrition status among vulnerable populations, we extracted 20 % of the poorest households and 20 % of the richest households from the overall population. We followed this by employing a multivariate regression where we found that residing in urban areas and children whose mothers had secondary schooling and higher were protective factors for children among the poorest households after controlling for wealth score. Also, urbanisation has both a direct and an inverse association with child mortality among sub-Saharan African countries, and the DRC, in particular, has experienced the highest reduction in child mortality as a result of urbanisation^(51)^. This finding is consistent with the reduction in child malnutrition in our study. The benefits of residing in urban areas include the availability of various food products, better infrastructure and more accessible healthcare resources due to the concentration of health workers^(48)^. Besides, the mother’s education has shown to be an independent protective factor for the nutrition status of the children living in the poorest households. Moreover, previous literature emphasised the importance of a mother’s education on children’s health and nutrition^(52,53)^. The various pathways through which a mother’s education promotes a child’s nutrition status include the acquisition of health knowledge, adherence to recommended feeding practices for children and increased command over resources.

There are some limitations to this study. Firstly, although we found significant associations between child malnutrition with geographical and socio-economic inequalities in this study, causal inference cannot be drawn as this was a cross-sectional study. Secondly, healthcare access is surrogated by maternal healthcare coverage in this study. This was due to the lack of available data to measure a household’s access to healthcare, such as distance to the closest clinic and affordability of medical visits. The study also assumed a linear additive relationship between the explanatory and dependent variables. The possibility of a non-linear relationship was not explored in our study. Finally, the household’s wealth status was measured based on asset endowments only.

## Conclusion

This study revealed considerable socio-economic inequalities in nutritional status among children in the DRC and the prevalence of malnutrition is higher in underdeveloped regions.

It calls for an urgent need for nutrition promotion in integrated services of maternal and child healthcare. Policy implementation of better healthcare should particularly prioritise central provinces and improve nutrition education for less-educated mothers.

## Supplementary material

For supplementary material accompanying this paper visit https://doi.org/10.1017/S1368980021004249. click here to view supplementary material
