# Supplementary material for: Socio-economic inequalities in children’s nutritional status in Democratic Republic of the Congo in 2017–2018: an analysis of data from a nationally representative survey
Source: Public Health Nutr. 2021 Oct 7;25(2):257–68. doi: 10.1017/S1368980021004249 (PMC8883769; doi:10.1017/S1368980021004249)
Supplement: Supplementary file 1 [file S1368980021004249sup001.docx]

Appendix: Weight-for-length/height z-score (WHZ) of children from the households of the lowest and highest wealth quintile

| WHZ | Lowest Wealth Quintile | | Highest Wealth Quintile | |
| --- | --- | --- | --- | --- |
|  | Coefficient | p-value | Coefficient | p-value |
|  | (95% CI) |  | (95% CI) |  |
| Maternal Healthcare Index | -0.015 | 0.878 | -0.070 | 0.601 |
|  | (-0.202, 0.173) |  | (-0.331, 0.192) |  |
| Domestic Sanitation Index | 0.151 | 0.015 | 0.000 | 0.997 |
|  | (0.030, 0.272) |  | (-0.134, 0.134) |  |
| Living in Rural | 0.182 | 0.179 | 0.912 | 0.018 |
|  | (-0.084, 0.448) |  | (0.154, 1.669) |  |
| Wealth Index | 0.550 | 0.019 | 0.270 | 0.029 |
|  | (0.092, 1.007) |  | (0.028, 0.512) |  |
| Mother’s Education (Reference: Illiteracy) | |  |  |  |
| Primary School | 0.290 | 0.046 | -0.261 | 0.152 |
|  | (0.006, 0.575) |  | (-0.619, 0.097) |  |
| Secondary School 1 | 0.192 | 0.429 | -0.420 | 0.047 |
|  | (-0.285, 0.669) |  | (-0.834, -0.006) |  |
| Secondary School 2 | 0.486 | 0.003 | -0.485 | 0.005 |
|  | (0.160, 0.812) |  | (-0.825, -0.144) |  |
| Above Secondary School | -0.815 | 0.081 | -0.069 | 0.778 |
|  | (-1.730, 0.100) |  | (-0.548, 0.410) |  |
| *Estimations are also adjusted for household size, child’s age (months), weight at birth(kg), child’s sex, and mother's age (years). | | | | |
